# Machine learning predicts nucleosome binding modes of transcription factors

**DOI:** 10.1186/s12859-021-04093-9

**Published:** 2021-03-30

**Authors:** K. C. Kishan, Sridevi K. Subramanya, Rui Li, Feng Cui

**Affiliations:** 1 Thomas H. Gosnell School of Life Sciences, Rochester Institute of Technology, 1 Lomb Memorial Drive, Rochester, NY 14623 USA; 2 Golisano College of Computing and Information Sciences, Rochester Institute of Technology, 20 Lomb Memorial Drive, Rochester, NY 14623 USA

**Keywords:** Machine learning, Nucleosome binding modes, Transcription factors

## Abstract

**Background:**

Most transcription factors (TFs) compete with nucleosomes to gain access to their cognate binding sites. Recent studies have identified several TF-nucleosome interaction modes including end binding (EB), oriented binding, periodic binding, dyad binding, groove binding, and gyre spanning. However, there are substantial experimental challenges in measuring nucleosome binding modes for thousands of TFs in different species.

**Results:**

We present a computational prediction of the binding modes based on TF protein sequences. With a nested cross-validation procedure, our model outperforms several fine-tuned off-the-shelf machine learning (ML) methods in the multi-label classification task. Our binary classifier for the EB mode performs better than these ML methods with the area under precision-recall curve achieving 75%. The end preference of most TFs is consistent with low nucleosome occupancy around their binding site in GM12878 cells. The nucleosome occupancy data is used as an alternative dataset to confirm the superiority of our EB classifier.

**Conclusions:**

We develop the first ML-based approach for efficient and comprehensive analysis of nucleosome binding modes of TFs.

**Supplementary Information:**

The online version contains supplementary material available at 10.1186/s12859-021-04093-9.

## Background

The nucleosome is the basic repeating structural and functional unit of chromatin, which consists of 147 base pairs of DNA wrapped around eight histones. Although nucleosomes cover most of the genome, their locations on DNA are not random. A long-standing goal in chromosome biology is to understand which factors control nucleosome positioning and how nucleosomes interact with those factors to regulate gene expression. One of the determinants of nucleosome positioning is transcription factors (TFs) including activators and components of the preinitiation complex [1].

The traditional view on nucleosome-TF interactions is that TFs displace nucleosomes to gain access to their cognate binding sites. The binding of TFs to a nucleosome results in a ternary structure that is relatively unstable [2] because the TFs have higher binding affinities to free DNA than nucleosomal DNA. This difference in binding affinities leads to the destabilization of the ternary structure. On the other hand, a subgroup of TFs, known as pioneer TFs [3], can interact with nucleosomal DNA, open chromatin, and establish developmental competence. We and other groups have shown that the rotational setting of binding sites in a nucleosome is a critical determinant for their accessibility to these pioneer TFs [4–6]. These studies show that pioneer TFs are able to bind nucleosomal DNA while most TFs appear not to have this ability.

A recent study systematically explored the modes of interactions between nucleosomes and 220 TFs that represent diverse structural families [7]. This study has identified several binding modes for the TFs including gyre spanning (GS), oriented binding (OB), end binding (EB), periodic binding (PB) and dyad binding (DB). Importantly, these modes are not mutually exclusive, meaning that a TF may have multiple nucleosome binding modes. These data clearly show that the binary classification of TFs based on their ability to bind nucleosomal DNA is not enough to capture the diversity of the interaction landscape between TFs and the nucleosome.

However, there are substantial experimental challenges to determine nucleosome binding preferences for thousands of TFs in different species. Thus, efficient computational methods capable of determining the preferences are needed. Here, we present ProtGauss, a machine learning (ML) model to predict nucleosome binding modes of TFs based on Gaussian representation for protein sequences. Our model differs from other ML methods by (1) using the Gaussian representation of sequences to capture the diversity of the subsequence representations via a covariance matrix and (2) designing a kernel function to capture the similarity between sequence features represented by Gaussian distributions. These differences are important because general ML methods often take vector representations of the sequences as input, which is obtained by computing the average of subsequence features from ProtVec [8]. The average representation however fails to capture the variation of subsequence features, and therefore is not expressive enough to represent an arbitrarily long protein sequence.

In this study, we focused on 167 TFs that have at least one nucleosome binding mode (or label) measured experimentally (see “Methods”). We used nested cross-validation to train and evaluate the model. The inner cross-validation is used to optimize the hyperparameters of the models and the outer cross-validation estimates the performance of the model with optimal hyperparameters. The nested cross-validation eliminates the bias introduced by simple cross-validation and can thus alleviate the overfitting problem. In this multi-label classification problem, the ProtGauss model outperformed several fine-tuned off-the-shelf ML methods including logistic regression, support vector machine, k-nearest neighbours, and random forest.

Then we built binary classifiers for individual binding modes and found that the classifier for the EB mode is the best. Our EB classifier was superior to the ML methods with the area under precision-recall (minor-AUPR) curve achieving 75%. We further showed that the EB mode of TFs is related to decreased nucleosome occupancy around their bind sites in GM12878 cells mapped by ChIP-seq (ChIP-sequencing) and MNase-seq (micrococcal nuclease digestion with deep sequencing). The nucleosome occupancy profiles around TF binding sites were used as an alternative dataset to confirm the superiority of the EB classifier compared to other ML methods.

Using the EB classifier, we predicted that a vast majority (88–99%) of TFs in five model organisms (yeast, nematode, fruit fly, mouse and human) have this binding mode with mammalian TFs being the lowest and the yeast TFs being the highest. Our prediction showed that several TFs in the SOX family including SOX2 and SOX11 do not have the EB mode, consistent with experimental studies [9]. Overall, this work represents the first systematic analysis of nucleosome binding mode of TFs using a computational method.

## Material and methods

**Fig. 1:**
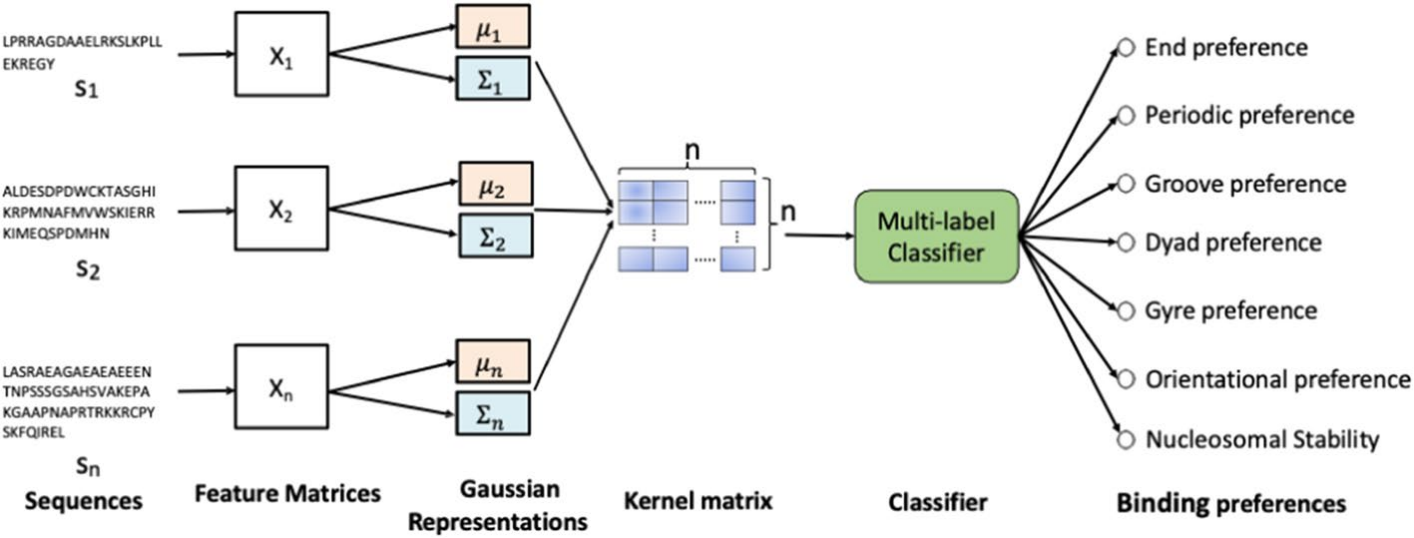
A model to predict nucleosome-TF binding patterns. The sequence $s_{i}$ is projected to a multivariate Gaussian distribution with mean $\tilde{\mu }_{i}$ and covariance matrix $\tilde{\Sigma }_{i}$. Similarity between these multivariate Gaussian distributions is computed to form kernel matrix and a multi-label classifier is trained to model binding preferences using the kernel matrix

Different components of the proposed machine learning method for predicting binding modes of transcriptions factors to nucleosomes are described below. Figure 1 shows the overall block diagram of ProtGauss. To learn feature matrix $X$, ProtVec [8] method is trained on the subsequences of fixed length $l_{s}$. Feature matrix $X$ is projected to mean vector $\mu$ and covariance matrix $\Sigma$ of Gaussian distribution. Then, the kernel matrix is defined using the similarity between Gaussian distributions and a multi-label classifier is trained to model nucleosome-binding preferences of TFs.

### Learning features from protein sequences

Amino acid sequences of TFs are the input to the proposed model. Breaking the sequences into fixed length overlapping subsequences (i.e. biological words) is the simplest and most common technique in bioinformatics to learn features [10, 11]. If the length of the sequence is $l$ and the length of the subsequence is $l_{s}$, the sequences can be broken into $l - \left( {l_{s} - 1} \right)$ subsequences. For simplicity, let the number of subsequences be represented by $L$.

**Fig. 2:**
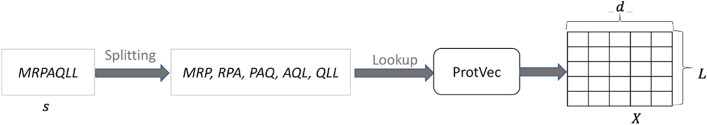
A workflow to represent an amino acid sequence as a matrix of ProtVec embeddings. A sequence $s_{i}$ is split into the subsequences with length $l_{s} = 3$ and the embeddings of these subsequences from the ProtVec model are used to obtain a feature matrix $X$

Given that a primary protein sequence can be split into overlapping subsequences of length $l_{s}$, we used ProtVec to extract features from the protein sequence. ProtVec embeds each subsequence to *d*-dimensional vector that characterizes the biophysical and biochemical properties of sequences. In particular, each subsequence of length $l_{s}$ is represented by a continuous vector of dimension $d = 100$. The parameter $d$ is a hyperparameter for ProtVec method. For the sequence that is split into $L$ subsequences, ProtVec model learns a matrix $X$ of shape $L \times d$ where each row represents the feature learnt for a subsequence. Figure 2 illustrates the details on how the sequences are projected to embedding matrix $X$ using ProtVec.

### Gaussian representation of sequences

A sequence of length $l$ is represented as the matrix $X$ of shape $L \times d.$ The feature matrix $X$ contains $d$ dimensional feature for $L$ subsequences. A simple approach to learn representation for sequences from the subsequence feature matrix $X$ is to compute the mean of the representation of subsequences [8]. However, the mean of the subsequence matrix blurs the representation of the subsequences and may not be good enough to represent the sequence. To address this challenge, we proposed a novel approach to represent the sequence $s$ as a multivariate Gaussian distribution and the biological words (subsequences) are assumed to be generated from that distribution (that is, the subsequence representations are the samples from this distribution) [12]. To the best of our knowledge, our proposed model is the first work to represent protein sequences as Gaussian distribution. Specifically, we consider the embeddings of all subsequences present in the sequence as the independent and identically distributed (i.i.d) samples drawn from the distribution:

1 $$x \sim N\left( {\mu , \Sigma } \right)$$

where $x$ represents the ProtVec representation for subsequence sampled from multivariate Gaussian distribution with mean $\mu$ and covariance matrix $\Sigma$.

Given the feature matrix $X$, the mean vector and the covariance matrix are set to their Maximum Likelihood estimates, given by the empirical mean $\tilde{\mu }$ and the empirical covariance matrix $\tilde{\Sigma }$ respectively. Specifically, the sample mean of the sequence corresponds to the mean of the subsequence representations, i.e. the vectors of the subsequences in the sequence are added and normalized by the number of subsequences. For a sequence $s$ with $L$ subsequences, the mean of the distribution is given by:

2 $$\tilde{\mu } = \frac{1}{L}\mathop \sum \limits_{i = 1}^{L} x_{i}$$

The empirical covariance matrix is then defined as:

3 $$\tilde{\Sigma } = \frac{1}{L}\mathop \sum \limits_{i = 1}^{L} (x_{i} - \tilde{\mu })(x_{i} - \tilde{\mu })^{T}$$

Since the sequence is represented as a multivariate Gaussian distribution with sample mean $\tilde{\mu }$ and the empirical covariance matrix $\tilde{\Sigma }$, the problem of classifying binding preferences based on the sequence transforms to classifying based on the distribution [12].

### Measuring similarity between sequences

To classify the multivariate Gaussian representation of protein sequences, we proceed by defining the similarity between the multivariate Gaussian distributions of two sequences for prediction of nucleosome binding modes. Here, the similarity measure between two distributions is proposed. The similarity between mean vectors $\tilde{\mu }_{i}$ and $\tilde{\mu }_{j}$ can be computed using cosine similarity as:

4 $$sim\left( {\tilde{\mu }_{i} , \tilde{\mu }_{j} } \right) = \frac{{\tilde{\mu }_{i} \cdot \tilde{\mu }_{j} }}{{||\tilde{\mu }_{i} ||_{2} ||\tilde{\mu }_{j} ||_{2} }}$$

where $\parallel \cdot \parallel_{2}$ denotes the Euclidean norm of the vectors. Similarly, the similarity between the covariance matrices $\widetilde{{\tilde{\Sigma }}}_{i}$ and $\tilde{\Sigma }_{j}$ can be computed as:

5 $$sim\left( {\tilde{\Sigma }_{i} , \tilde{\Sigma }_{j} } \right) = \frac{{\mathop \sum \nolimits_{{}}^{{}} \tilde{\Sigma }_{i} \odot \tilde{\Sigma }_{j} }}{{||\tilde{\Sigma }_{i} ||_{F} ||\tilde{\Sigma }_{j} ||_{F} }}$$

where $\odot$ is the element-wise multiplication between the matrices and $\parallel \cdot \parallel_{F}$ represents the Frobenius norm of the matrix. Then, the similarity between two sequences $s_{i}$ and $s_{j}$ is measured as the convex combination of the similarity between their mean vectors $\tilde{\mu }_{i}$ and $\tilde{\mu }_{j}$ and their covariance matrices $\tilde{\Sigma }_{i}$ and $\tilde{\Sigma }_{j}$. Therefore, the similarity between two sequences $s_{i}$ and $s_{j}$ is given by:

6 $$sim\left( {s_{i} , s_{j} } \right) = \alpha \cdot sim\left( {\tilde{\mu }_{i} , \tilde{\mu }_{j} } \right) + \left( {1 - \alpha } \right) \cdot sim\left( {\tilde{\Sigma }_{i} , \tilde{\Sigma }_{j} } \right)$$

where $\alpha \in \left( {0, 1} \right)$ is the hyperparameter that controls the relative importance of similarity between mean vectors and the similarity between the covariance matrices. A kernel matrix $K$ is defined where $K_{ij} = sim\left( {s_{i} , s_{j} } \right)$.

### Training

Training our proposed model involves two steps: (a) training ProtVec to learn representation of protein sequences and (b) converting these representations to Gaussian distributions and training Support Vector Machine (SVM) with similarity kernel between Gaussian distributions. First, we collected 561,568 sequences from Swiss-Prot database (UniProt release 2019_11) and trained the ProtVec model on these sequences. The trained ProtVec model was used to obtain the feature matrix $X_{i}$ for each sequence $s_{i}$. Second, the Gaussian representation for each sequence is obtained from their respective feature matrix and the similarities between sequences is computed to define the kernel for Support Vector Machine (SVM) [13]. Since the TFs can have multiple binding preferences, it is a multi-label classification task. One-vs-Rest classifier is used to train on multi-label classification problems. We used the scikit-learn library in Python to implement the SVM classifier and nested cross-validation with 10 outer and 10 inner cross-validation to report the results.

The two metrics were considered for performance evaluation of our proposed method as well as other baselines in this task. (1) Accuracy is also known as subset accuracy, that measures the percentages of test TFs that were correctly predicted (i.e. a TF is correctly predicted if the set of predicted binding preferences of this TF exactly matches with the set of its ground-truth preferences; in this case, the ground-truths are nucleosome binding preferences of TFs that are measured experimentally [7]). (2) Micro-averaged area under the precision-recall curve (micro-AUPR) [14] combines the predictions across all binding preferences into a vector, and then the area under the precision-recall curve is computed based on that vector. We further considered Matthews correlation coefficient (MCC) as a measure of the quality of binary classifications to compare several methods with nucleosome occupancy data. This metric addresses the concern of an imbalanced testing set and obtains more reliable performance. MCC can be computed as

7 $$MCC = \frac{TP \times TN - FP \times FN}{{\surd \left( {TP + FP} \right)\left( {TP + FN} \right) \left( {TN + FP} \right)\left( {TN + FN} \right)}}$$

For multi-label classification, ranking-based metrics such as micro-AUPR is an appropriate metric for class imbalance scenarios [15]. In this work, the class imbalance is extreme i.e. the EB mode has 121 positives, the gyre spanning mode has only 3 positives, the dyad binding mode has 10 positives, and the orientational binding mode has 12 positives out of 167 TFs (Table 1). Since micro-AUPR gives equal importance for all samples across classes, classes with relatively few positive samples should not influence the overall AUPR score of the model if the model is performing well on other common binding preferences.

**Table 1:** Datasets used in the study

Binding preferences	No. of positive samples	No. of negative samples
End preference	121	46
Periodic preference	98	69
Groove preference	45	122
Dyad preference	10	157
Gyre spanning	3	164
Orientational preference	12	155
Nucleosome stability	71	96

When we formulate multi-label classification as multiple binary classification, accuracy is not an appropriate metric to compare different models. For example, there are 10 positive and 157 negative cases for the dyad binding mode. A simple majority classifier predicts all the TFs to have no dyad preference, the accuracy of the classifier will be 94.01% (= 157/167). In contrast, PR curves are appropriate to classify such binding preferences and therefore we chose micro-AUPR for comparison.

### Baselines

For the baseline methods, the mean of the feature matrix $X \in R^{L \times d}$ from ProtVec was taken across subsequences as: $x = \frac{1}{L}\sum\nolimits_{i}^{L} {X_{i} \in R^{d} }$ to obtain the feature vector. The feature vector $x \in R^{d}$ is used to train other baselines for performance comparison. Our proposed model was compared with Logistic Regression (LR), Support Vector Machine (SVM) [13], K-nearest neighbours (kNN) [16], and Random Forest (RF) [17, 18]. All of these methods were implemented using the scikit-learn library in Python. Table 2 shows the list of settings for tuning parameters of these baselines. We adopted grid search with nested cross-validation to find the optimal parameters and evaluate the performance of the baselines.

**Table 2:** The parameters and set of values for various off-the-shelf baselines

Method	Tuning parameters
Logistic regression	The norm used in the penalization: none, L1, L2, elastic net Regularization coefficient: 100, 10, 1, 0.1, 0.01 Subsequence length: 3, 4, 5, 6
k-nearest neighbors	Number of neighbors to use: 1, 3, 5, 7, 9, 11, 13, 15, 17, 19, 21 Contribution of members in the neighborhood: uniform, distance Distance metric: Euclidean, Manhattan, Minkowski Subsequence length: 3, 4, 5, 6
Support vector machine	Kernel: Linear, Polynomial, RBF, Sigmoid Regularization parameter (C): 50, 10, 1.0, 0.1, 0.01 Subsequence length: 3, 4, 5, 6
Random forest	The number of features to consider when looking for the best split: sqrt(num features), log2(num features) The number of trees in the forest: 10, 100, 200, 500, 1000 Subsequence length: 3, 4, 5, 6

### Datasets

#### TF datasets

Seven nucleosome-TF interaction patterns for 195 TFs from diverse structural families were determined experimentally in a prior study (Table S5 in [7]). The interaction modes are not mutually exclusive, and a given TF can have more than one binding modes. These experimentally determined binding modes are used as ground truths of the present study. For multi-label classification, it is important for TFs to belong to at least one class. However, there are 28 TFs that have none of these patterns. Thus, 167 (= 195 − 28) TFs have at least one binding mode. Moreover, 24 TFs have ChIP-seq data from GM12878 cells in ENCODE that were used for testing the model (see below) and 21 of them belong to the 167 TFs. As a result, the remaining 146 (= 167 − 21) TFs were used to train the model (Additional file 1: Supplementary Table S1). The full-length sequences of TFs were taken from Animal TFDB 3.0 [19].

The tested ProtGauss model was applied to all TFs from five model species, including 1,664 TFs from human (Additional file 1: Supplementary Table S2), 1,636 TFs from mouse (Additional file 1: Supplementary Table S3), 651 TFs from fruit fly (Additional file 1: Supplementary Table S4), 748 TFs from nematode (Additional file 1: Supplementary Table S5), and 296 TFs from yeast (Additional file 1: Supplementary Table S6). The full-length sequences of the TFs, except those from yeast, were downloaded from Animal TFDB 3.0 [19] in the FASTA format. The human TF protein sequences file contained a total of 1,675 sequences which included repeated TF entries for “HOPX” (10 times), “ZNF177” (2 times), and “LCOR” (4 times). Note that HOPX and LCOR each have two unique sequences. Thus, these three TFs have five unique sequences that were retained. The remaining entries (11 sequences) were omitted, which results in the human dataset containing 1664 (= 1675 − 11) TF sequences. Among these 1,664 TFs, two sequences have no TF name. Their names (H3BRB8/H3BSE6 and PAWR) were extracted using the Ensembl ID provided in the fasta file. The DNA-binding domains of the TFs were also retrieved from the same file. However, some of the entries had "Miscellaneous" as a domain. Thus, Pfam batch sequence search [20] utility from EMBL-EBI was used to determine the unknown TF domains in batch. The same process was incorporated to extract the unknown TF domains in the other 4 species. Finally, yeast DNA-binding TFs were taken from literature [21] and their full-length sequences were retrieved from UniProt.

#### Nucleosome (MNase-seq) datasets

The MNase-seq short reads for in vivo nucleosomes in the GM12878 cell line (hg19) were downloaded from the University of California Santa Cruz (UCSC) Genome Browser HTTP server [22]. A total of nine BAM files that are in Gene Expression Omnibus (GEO) ID GSM920558 with names wgEncodeSydhNsomeGm12878AlnRepX.bam, where X is between 1 and 9 were downloaded, merged, and sorted based on chromosomes using SAMtools [23]. The reads in each chromosome file were extended to 147 bp in the 5′ to 3′ direction.

The normalized nucleosome occupancy of a TF was calculated at each nucleotide position in the genome by dividing the total number of nucleosomal DNA sequences surrounding the ChIP peak position by the average number of nucleosomal sequences across the genome as described in the paper [24]. The normalized values were smoothed with a 61-bp window.

#### ChIP-seq datasets

Out of the 1,664 human TFs, 106 TFs have ChIP-seq data available in the GM12878 cell line, which were deposited in ENCODE database [25]. The GM12878 cell line was chosen because (1) it is a Tier 1 cell line in the ENCODE project in which a number of ChIP-seq datasets are available and (2) it is derived from normal cells that have no global aberrant epigenetic modifications and chromatin reorganization, which are often observed in cancer chromatin [26].

The ChIP-seq peaks (i.e., the ‘optimal’ set) of the 106 TFs in the human genome hg19 were downloaded from ENCODE. Among 106 TFs, 24 TFs have known E-MI penetration (lig147) values [7] (Additional file 1: Supplementary Table S7) and 82 TFs do not have E-MI values (Additional file 1: Supplementary Table S8). E-MI stands for enriched-sequence-based mutual information, which provides information about the relative location of TF binding in nucleosomal DNA [7]. TFs with E-MI < 20 indicate that the TFs prefer to bind nucleosome ends, whereas TFs with E-MI > 20 indicate that the TFs do not have this preference. Note that there is no overlap between the 106 TFs with the 146 TFs used to train the ProtGauss model. The overlap between the 106 TFs and the 195 TFs with E-MI values [7] is the set of 24 TFs that was used as the test set.

### Calculation of nucleosome occupancy around TF binding sites

For a given TF with ChIP-seq data available in the GM12878 cell line, the nucleosome occupancy profile was calculated around the ChIP peak centre (± 1000 bp). Two groups of human TFs in GM12878 cells were used to calculate the nucleosome occupancy profiles: one contains 24 TFs with both ChIP-seq data and known EMI penetration (lig147) values (Additional file 1: Supplementary Table S7), and the other includes 82 TFs with ChIP-seq data but not EMI penetration values (Additional file 1: Supplementary Table S8). For TFs in both groups, the nucleosome occupancy patterns around ChIP peak center were divided into three types: ‘peak-at-centre’, ‘dip-at-centre’ and ‘questionable’ (see detailed below). Note that 5 TFs have the ‘questionable’ pattern (Additional file 1: Supplementary Table S8), resulting in 101 (= 24 + 82 − 5) TFs that have either ‘peak-at-centre’ or ‘dip-at-centre’ nucleosome occupancy patterns (Additional file 1: Supplementary Table S7 and S8).

## Results

### Extensive exploration of subsequence length $l_{s}$

ProtVec method learns the representation of sequence by breaking the sequence into subsequences (i.e. biological works) of length $l_{s}$. The length $l_{s}$ is a hyperparameter that plays a key role in learning representation of the protein sequences and thus significantly impacts the performance of proposed model that uses features learned by ProtVec as input.

**Fig. 3:**
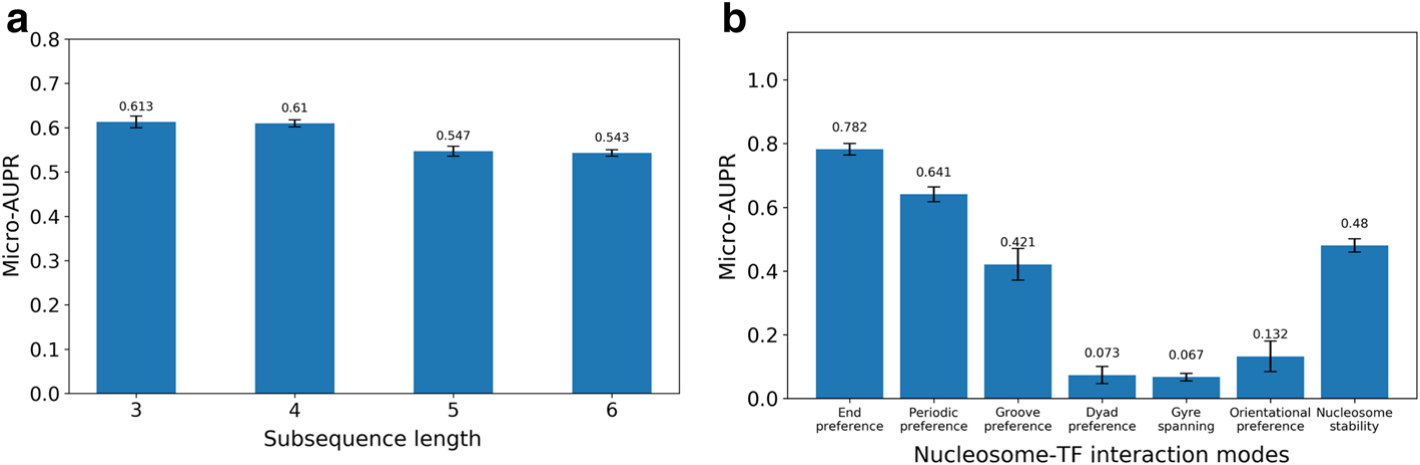
Performance comparison of the model trained on full-length sequences of TFs. **a** Micro-AUPR and accuracy comparison between models trained on all binding patterns with different subsequence lengths. **b** Micro-AUPR and accuracy comparison between models trained with subsequences with the length of 4 residues for individual binding patterns

Thus, to determine the optimal subsequence length $l_{s}$, the impact of the subsequence length on the performance of the proposed model was systematically evaluated. This is because the length of subsequence plays an important role to learn the representation using ProtVec (see “Methods”). For this experiment, two different settings were considered for subsequence lengths of 3, 4, 5, or 6 amino acids. First, multi-label classification was performed to compare various subsequence lengths (Fig. 3a). Second, binary classification was performed for individual binding modes (i.e., DB, EB, GB, OB and PB, as well as nucleosome stability) across subsequence lengths (Fig. 3b, Additional file 2: Supplementary Figure S1-S3). For both experiments, micro-AUPR was used as a comparison metric since it indicates how the model performs overall for all binding preferences and is not sensitive to the predictive performance for individual binding preferences.

For the multi-label classification task, the model trained with the subsequence length $l_{s} = 4$ achieved superior performance compared to other subsequence lengths. In particular, the model trained using $l_{s} = 4$ achieved 0.61 on micro-AUPR and 0.224 on accuracy. The model trained with $l_{s} = 3$ achieved similar performance i.e. 0.613 on micro-AUPR and 0.176 on accuracy. Furthermore, we observed that using longer subsequence length (5 or 6) substantially decreases the performance of the model (Fig. 3a).

For the binary classification task, among the five binding modes and nucleosome stability, we observed that the classifier performed best for the EB mode compared to other nucleosomes-binding modes (Fig. 3b, Additional file 2: Supplementary Figure S1–S3). The performance of the binary classifiers generally follows a decreasing trend with increasing subsequence lengths from 3 to 6, in which the classifier with subsequence length 4 outperforms other subsequence lengths (Fig. 3b, Additional file 2: Supplementary Figure S1–S3). Thus, we selected the subsequence length $l_{s} = 4$ for the following experiments.

### Optimization of the hyper-parameter $\alpha$

**Fig. 4:**
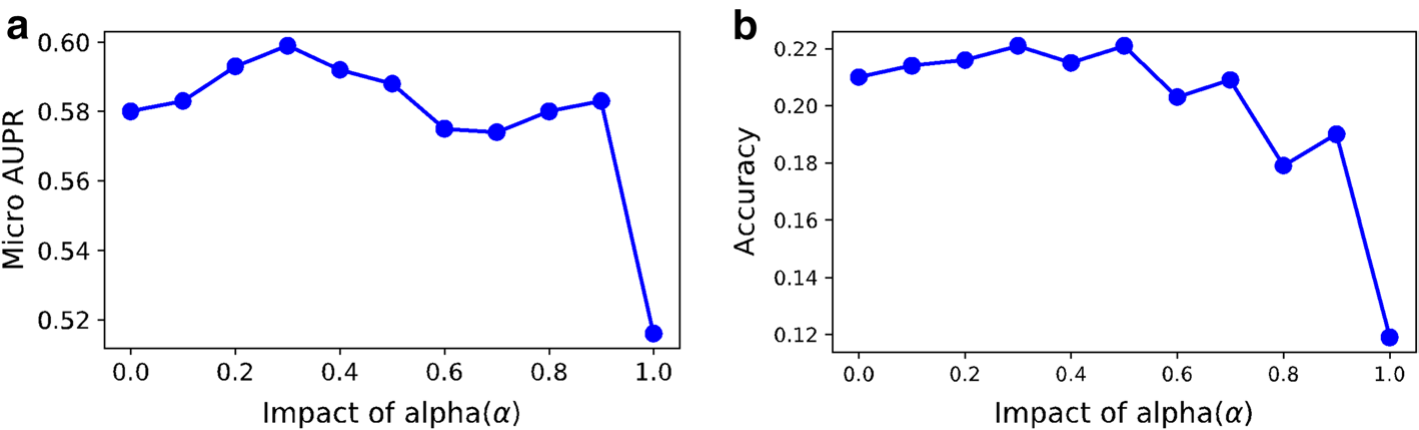
Impact of $\alpha$ on the performance of proposed methods measured by various performance metrics such as **a** micro-AUPR, **b** accuracy. $\alpha \in \left[ {0, 1} \right]$ controls the impact of similarity between mean vectors and covariance matrices. If $\alpha = 0$, only the similarity between covariance matrices is considered. In contrast, the only similarity between mean vectors is considered when $\alpha = 1$

To optimize the hyper-parameter $\alpha$ in Eq. 6, the impact of different $\alpha$ values on the performance of our model was measured by two metrics, micro-AUPR (Fig. 4a) and accuracy (Fig. 4b). An appropriate value of $\alpha$ that controls the relative importance of the mean and covariance matrix is crucial for our model. For this experiment, the model was trained with subsequence length $l_{s} = 4$. The model achieved the best micro-AUPR when the value of $\alpha$ is 0.3. Furthermore, when the similarity between covariance matrices was removed (with $\alpha$ = 1), our method considers only the similarity between the mean vectors of sequences and the performance dropped significantly (Fig. 4a, b). These results support the idea of representing the protein sequences as Gaussian distributions instead of representing them as mean vectors of subsequence representation.

### Comparison between ProtGauss and off-the-shelf methods on binding preferences of TFs

To evaluate the performance of our model on all nucleosome-binding preferences of a given TF, we compared it with four off-the-shelf ML methods that are used as baselines (see “Methods”). Because the off-the-shelf ML methods are often not tuned for our prediction tasks, to get a fair comparison with our model, we fine-tuned the baselines using a set of parameter values (Table 2). For this experiment, we used subsequence length $l_{s} = 4$ together with $\alpha$ = 0.3 for our method, and compared with other methods trained with optimized parameters. We found that our ProtGauss model outperformed the baselines in two metrics, micro-AUPR, and accuracy (Table 3). Specifically, our model achieved 0.61 on micro-AUPR and 0.224 on accuracy. By contrast, an optimized SVM model rendered 0.544 on micro-AUPR and 0.155 on accuracy. Compared with the SVM model, the ProtGauss model achieved improvements of 12.1% on micro-AUPR, and 44.5% on accuracy. The results showed that the proposed similarity-based kernel achieves significant improvement over the SVM classifier that does not consider the variance of the features. Overall, we demonstrated the effectiveness of our proposed method and the benefit of using multivariate Gaussian distributions to represent sequences for measuring similarity between a pair of sequences.

**Table 3:** Performance comparison of our proposed method (in bold) with other baselines for all binding mode data

Method	m-AUPR	Accuracy
LR	0.536 ± 0.013	0.129 ± 0.011
kNN	0.532 ± 0.007	0.132 ± 0.012
SVM	0.544 ± 0.018	0.155 ± 0.026
RF	0.558 ± 0.022	0.157 ± 0.016
**ProtGauss**	**0.61** ± **0.008**	**0.224** ± **0.012**

### Binary classifier for the EB mode outperforms those for other binding modes

To identify which individual binding mode the ProtGauss model performs the best, we built a binary classifier for each binding mode and nucleosome stability. The performance of the classifiers trained on the full-length sequences of TFs was evaluated with the subsequence length $l_{s} = 4$ together with $\alpha$ = 0.3. It has been shown that the highest micro-AUPR was achieved for the EB mode (Fig. 3b), indicating that the ProtGauss classifier for the EB mode prediction outperforms the classifiers for other binding modes.

To check if the ProtGauss classifier for the EB mode is better than other fine-tuned ML models, we applied these models to the 24 TFs with E-MI data (Additional file 1: Supplementary Table S7). We found that our model achieved the highest micro-AUPR (0.75), accuracy (0.776) and MCC (0.352), compared to other models (Table 4). This result indicates that, in addition to the multi-label classification problem (Table 3), our model outperforms other models in the binary classification problem (Table 4).

**Table 4:** Performance comparison of the proposed method (in bold) and various baselines for end binding mode data

Method	m-AUPR	Accuracy	MCC
SVM	0.739	0.764	0.322
RF	0.718	0.713	0.122
LR	0.738	0.726	0.269
KNN	0.716	0.709	0.075
**ProtGauss**	**0.75**	**0.776**	**0.352**

### End preference of TFs is related to low nucleosome occupancy around TF binding sites

For a given TF, its genome-wide binding sites are measured by ChIP-seq assays, whereas nucleosome locations across the genome can be determined by MNase-seq assays. Nucleosome occupancy reflects the fraction of cells from a population in which a given region of DNA is occupied by a histone octamer [1]. TF binding sites at nucleosomal DNA ends become more accessible due to a process known as “breathing” [27–29], in which DNA is detached from histones. Thus, if a TF preferentially binds to the ends of a nucleosome, its binding sites are likely to have a relatively lower nucleosome occupancy, compared to a TF that binds to the central region of a nucleosome.

**Fig. 5:**
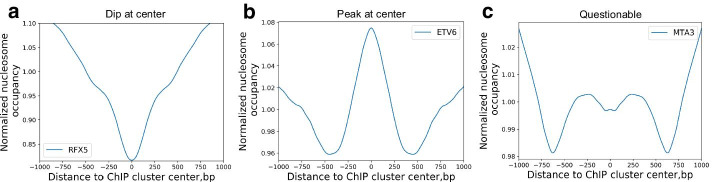
Profiles of nucleosome occupancy around **a** RFX5 ChIP clusters showing dip at centre (dip), **b** NFATC1 ChIP clusters showing peak at centre (peak), and **c** FOXK2 ChIP clusters showing no clear peak or dip at centre (questionable)

To link the nucleosome EB mode of TFs measured in vitro with nucleosome occupancy around TF binding sites measured in vivo, we assessed 106 TFs in GM12878 cells, which have both ChIP-seq and MNase-seq data (Additional file 1: Supplementary Table S7 and S8). For each TF, the average nucleosome occupancy was calculated for the genomic regions around the centre of ChIP fragments. Three types of nucleosome occupancy profiles were identified: (1) dip at centre (Fig. 5a); (2) peak at centre (Fig. 5b); and (3) questionable, in which no clear dip or peak is shown (Fig. 5c). Out of the 106 TFs, 86, 15 and 5 TFs has the ‘dip-at-centre’, ‘peak-at-centre’ and ‘questionable’ profiles, respectively (Additional file 1: Supplementary Table S7 and S8).

**Fig. 6:**
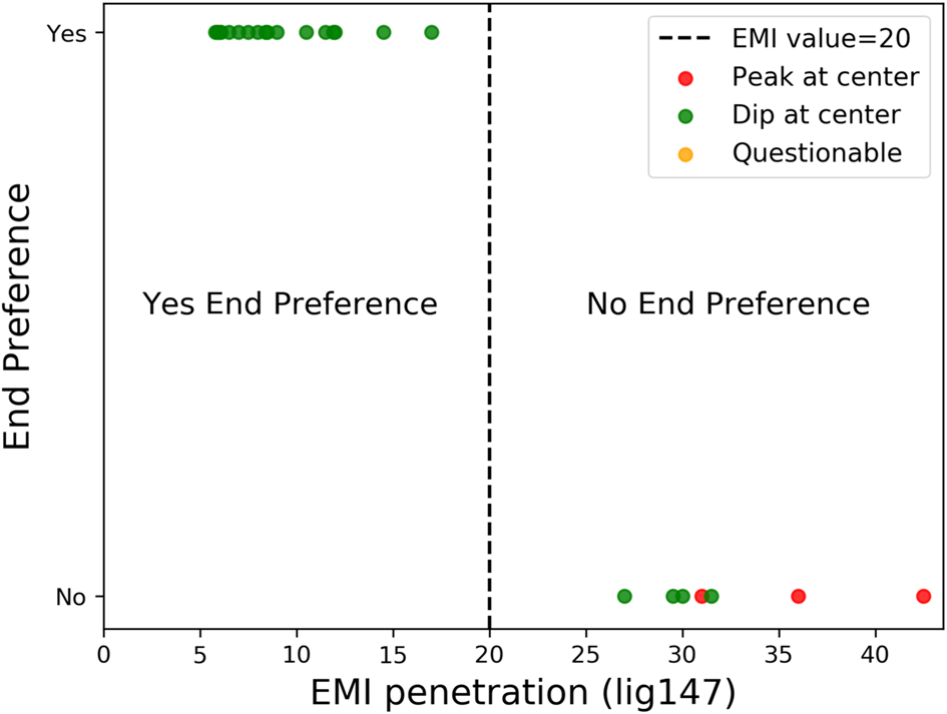
Categorization of nucleosome occupancy profiles for 24 TFs with known E-MI penetration (lig147), in terms of peak (red), dip (green), and questionable (orange) nucleosome occupancy profiles. TFs with an E-MI penetration (lig147) less than 20 are defined as having end preference (7)

Detailed analysis of the 24 (out of 106) TFs with both ChIP-seq data and E-MI penetration (lig147) values (Additional file 1: Supplementary Table S7) revealed a clear trend (Fig. 6). That is, TFs with the EB mode, which have EMI penetration values < 20 as defined in [7], tend to have the ‘dip-at-centre’ nucleosome occupancy profile. By contrast, TFs without the end preference (with E-MI penetration values > 20) tend to have the ‘peak-at-centre’ nucleosome occupancy profile. The higher E-MI penetration value a TF has, the more likely it has the ‘peak-at-centre’ profile. Our data establish a clear correlation relationship between nucleosome occupancy profiles around TF binding sites and the EB mode of TFs. The nucleosome occupancy of TFs can be used as an alternative dataset to test our ProtGauss model as well as other baselines.

### Use of nucleosome occupancy data as an alternative test dataset

To further illustrate our ProtGauss model outperforms fine-tuned baselines, we used the 86 TFs with the ‘dip-at-centre’ profile and 15 TFs with the ‘peak-at-centre’ profiles as an alternative test set. The 5 TFs with the ‘questionable’ profile are not included. Note that the 101 (= 86 + 15) TFs are not in the training set. The performance on this test set indicates the ability of various models to be generalized to new sequences that were not the part of the training procedure. We found that our model is superior to other models in the alternative test set (Table 5). We also provided a summary of prediction results on the nucleosome occupancy data as a confusion matrix (Table 6). Specifically, our method identified more true positives and true negatives combined (i.e., 85 + 3 = 88) and less false positives and false negatives combined (i.e., 1 + 12 = 13), compared to other methods. The second best model is KNN (Table 5). It identifies all TFs to have end preference; it has 86 true positives and true negatives combined (i.e., 86 + 0 = 86) and 15 false positives and false negatives combined (i.e., 15 + 0 = 15). Furthermore, random forest achieves similar performance to kNN but identifies 81 true positives and true negatives combined (i.e., 78 + 3 = 81) and 20 false positives and false negatives combined (i.e. 8 + 12 = 20). These results indicate that our ProtGauss model not only correctly predicts end binding modes of TFs but also reduces the number of incorrect predictions.

**Table 5:** Performance comparison of proposed method (in bold) and other baselines for nucleosome occupancy data

Method	m-AUPR	Accuracy	MCC
SVM	0.864	0.822	-0.073
RF	0.86	0.851	0.14
LR	0.863	0.733	0.072
KNN	0.863	0.851	0.14
**ProtGauss**	**0.898**	**0.871**	**0.34**

**Table 6:** Comparison of confusion matrix for proposed method (in bold) and other baselines on nucleosome occupancy data

Method	True positives	True negatives	False negatives	False positives
SVM	76	2	10	13
RF	78	3	8	12
LR	68	4	18	11
KNN	86	0	0	15
**ProtGauss**	**85**	**3**	**1**	**12**

### Predicted nucleosome-binding modes in TFs of eukaryotes

To gain insights into the EB modes of eukaryotic TFs, we applied the ProtGauss model to thousands of TFs in five model species including yeast, nematode, fruit fly, mouse and human (Table 7). Examination of the fraction of TFs with predicted end preference reveals an interesting trend in eukaryotes. That is, the fraction of TFs with predicted EB mode achieves the highest in yeast (98.98%) and becomes lower in higher eukaryotes such as nematode (95.59%), fruit fly (96.01%), mouse (86.43%) and human (88.34%). This result indicates that compared to yeast, higher eukaryotes like mammals contain more TFs that do not have end binding preference and potentially target the central region of nucleosomes.

**Table 7:** Summary of EB mode prediction for transcription factors (TF) in different species

Species	Predicted end preference		Total
	Yes	No	
Human	1470 (88.34%)	194 (11.66%)	1664
Mouse	1414 (86.43%)	222 (13.57%)	1636
Fruit fly	625 (96.01%)	26 (3.99%)	651
Nematode	715 (95.59%)	33 (4.41%)	748
Yeast	293 (98.98%)	3 (1.01%)	296

### Human TFs not having end preference are enriched in the SOX and HOX families

To characterize the human TFs with no EB preferences, we first focused on the 13 pioneer TFs [3] that are capable of binding nucleosomal DNA (Additional file 1: Supplementary Table S9). It was found that 9 out of the 13 TFs are predicted to have end preference including OCT4/POU5F1 and p53. This result is consistent with previous studies that these two proteins interact with the end of nucleosomal DNA [30, 31].

A detailed analysis of the 1,664 human TFs showed that the TFs from a protein family are likely to share the same DNA-binding domain (Additional file 1: Supplementary Table S10). Grouping the TFs based on their DNA-binding domains rendered 74 unique domain families, in which 10 domain families have at least 20 TFs (Additional file 1: Supplementary Table S11). Analysis of these 10 domain families revealed that the HMG domain (52 TFs) and the homeobox domain (198 TFs) stand out, with 11 and 67 TFs not having the EB mode, respectively (Additional file 1: Supplementary Table S12).

Further analysis showed that the SOX family, one of HMG-containing TF families, contains a large fraction of TFs (10 out of 19, 53%) not having the end preference, including SOX2 and SOX11 (Additional file 1: Supplementary Table S12). This result is consistent with the cryo-electron microscopy (cryo-EM) structure of SOX2 or SOX11 in complex with a nucleosome, in which SOX2 and SOX11 interact with nucleosomal DNA at the superhelical location 2, which is close to the center (dyad) of the nucleosome [9]. On the other hand, TFs without the EB mode are also enriched in the HOX family, one of the homeobox-containing TF families, with 23 out of 39 (59%) TFs having no end preference (Additional file 1: Supplementary Table S13). These data are in accordance with recent work showing that the HOX family proteins have strong binding selectivity to less accessible chromatin regions [32]. Note that most members of two well-established pioneer TF families, FOX and GATA, are predicted to have the EB mode, with only 17 (out of 49) and 0 (out of 10) TFs not having the EB mode, respectively (Additional file 1: Supplementary Table S13), indicating that TFs from these two families tend to interact with the end of nucleosomal DNA.

## Discussion

In this paper, we develop a novel sequence-based machine learning model, ProtGauss, to predict nucleosome binding modes of TFs identified in previous studies [7]. Our model splits a protein sequence into overlapping $l_{s}$-length subsequences, and the embeddings of these subsequences are learned with the ProtVec model [8] to obtain a feature matrix. With extensive exploration of subsequence lengths, we found that the length of 4 amino acids is optimal for predicting nucleosome binding modes of TFs. We also tuned the hyper-parameter α to achieve a high performance. With the optimal subsequence length and α, our model outperformed four off-the-shelf machine learning methods.

For comparison with the four machine learning algorithms, we used two different metrics that capture the performance of the models from different perspectives. Accuracy is not appropriate for this task because of the imbalanced experimental data (Table 1). Also, accuracy is a strict metric that requires all the binding preferences to be predicted correctly to classify TFs as correctly classified. Thus, other metrics such as micro-averaged AUPR score are better alternatives for imbalanced data and multi-label classification. Moreover, micro-AUPR is used to compare the performance of different configurations of our models because micro-AUPR is not sensitive to the performance of the model on individual classes. Furthermore, in case of binary classification, we used MCC to compare models for predicting end binding preference. Since the number of positive samples are relatively larger than negative samples, micro-AUPR may be biased and may lead to overestimated performance for binary classification.

With the Gaussian representation of the sequences and the multi-label binding preferences, we adopted nested cross-validation to train and evaluate our proposed approach. We note that our model is robust with the random splitting of the dataset into folds over multiple runs. Furthermore, nested cross-validation allows us to tune the hyper-parameters using inner cross-validation and evaluate the performance of optimized models using outer cross-validation to alleviate the problem of overfitting.

**Fig. 7:**
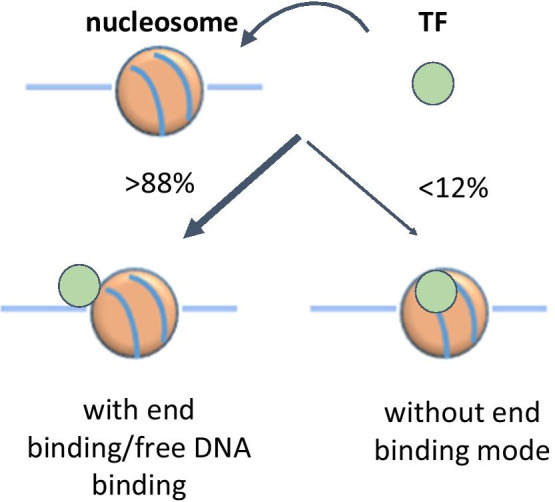
A model for the end binding mode of TFs to a nucleosome. Over 88% of TFs in five model species are predicted to bind nucleosomal DNA ends or free DNA, whereas less than 12% of the TFs are predicted not to have the end binding preference. The fraction of the TFs binding to nucleosomal DNA ends or free DNA is the highest in yeast (~ 99%) and the lowest in mammals (~ 88%)

We built binary classifiers based on full length sequences of TFs to identify which binding mode our method works best, and found that the classifier for the EB mode is superior to other classifiers. Testing this classifier with 24 TFs in the test set and 101 TFs in the alternative test set based on nucleosome occupancy data showed that the ProtGauss model outperformed 4 baselines (Tables 4, 5). We further applied the classifier for end preference to thousands of TFs in five model organisms. Based on the results, we proposed a model for the EB mode of TFs (Fig. 7). That is, more than 88% of all TFs are likely to bind to the ends of a nucleosome or free DNA. This model is consistent with a well-known observation that most TFs are located in genomic regions with low nucleosome occupancies [33]. Interestingly, the fraction of TFs with end preference is decreased in higher eukaryotes, compared to yeast. Furthermore, we found that most known pioneer factors have the EB mode, and the TFs having no end preference are enriched in the SOX and HOX protein families.

These observations have important implications. First, the number of TFs predicted not having the EB mode is increased in higher eukaryotes suggesting that these TFs may play an important role in differentiation and development. Second, the vast majority of known pioneer TFs (9 out of 13) have the EB mode, suggesting that the end preference is one of the main features of pioneer TFs. Third, many SOX and HOX family proteins have no end preference and can potentially target the central region of a nucleosome [7], suggesting that these TFs possess specific structural motifs that allow them to recognize cognate binding sites located inside of a nucleosome. It remains to be determined if they represent a new class of TFs that function differently from known pioneer TFs.

It is intriguing that some TFs (e.g., SOX14) are classified as having end-binding preferences and other TFs in the same protein family (e.g., SOX2 and SOX12) are predicted to bind internal sequence of a nucleosome. In our view, TFs in the same protein family may work in a coordinated manner. The TFs that are able to bind the central region of a nucleosome may act as a pioneer TFs to open chromatin. After that, this TF may be replaced by other family members that can only bind nucleosome ends or free DNA to initiate a developmental process. One example supporting this interpretation comes from the GATA family. GATA1/2/3 factors are required for the differentiation of mesoderm-derived tissues, including the hematopoietic system [34]. In particular, GATA2 is uniquely induced by BMP4 signaling during the establishment of hematopoietic stem/progenitor cells (HSPC) [35–37] and is suppressed during the differentiation of HSPC to proerythroblasts (ProE). This suppression is mediated by the displacement of GATA2 from its upstream enhancer by GATA1, a process referred to as the ‘GATA switch’ [38].

## Conclusions

ProGauss is a powerful machine learning method that learns features from protein sequences by mapping short subsequence representations as Gaussian distributions. The similarities between these distributions are used to define the kernel for training a SVM classifier. This method has been successfully applied to predict nucleosome binding modes of TFs, outperforming four other machine learning approaches. A binary classifier for the EB mode of TFs was applied to TFs in five model species, and it was found that about 88% of human TFs have this mode. Human TFs not having this mode are enriched in SOX and HOX TF families. Understanding whether these TFs have pioneering activities will shed new light on mechanisms underlying chromatin opening and developmental competence.

## Availability and requirements

- Project name: ProtGauss
- Project home page: https://github.com/kckishan/ProtGauss
- Operating system: Ubuntu 18.04.5 LTS (Bionic Beaver)
- Programming languages: Python3.6.9
- Other requirements: argparse 1.1, gensim 3.8.1, numpy 1.16.1, pandas 1.0.0, pickle 4.0, prettytable 0.7.2, pyfasta 0.5.2, scikit-learn 0.23.1, texttable 1.6.2, tqdm 4.40.2
- License: GNU General Public License

## Supplementary Information

**Additional file 1.** Supplementary Tables S1 to S13.

**Additional file 2.** Supplementary Figures S1 to S3.

## Data Availability

ProtGauss is available in the GitHub repository (https://github.com/kckishan/ProtGauss).

## Abbreviations

ML: Machine learning
Minor-AUPR: Area under precision-recall
TF: Transcription factor
EB: End binding
MCC: Matthews correlation coefficient
SVM: Support vector machine
