# Supplementary material for: Machine learning predicts nucleosome binding modes of transcription factors
Source: BMC Bioinformatics. 2021 Mar 30;22:166. doi: 10.1186/s12859-021-04093-9 (PMC8008688; doi:10.1186/s12859-021-04093-9)

**Supplementary Materials**

**Machine learning predicts nucleosome binding modes of transcription factors**

Kishan KC^2†^, Sridevi K. Subramanya^1†^, Rui Li^2^ and Feng Cui^1^*

^1^Thomas H. Gosnell School of Life Sciences, Rochester Institute of Technology, 1 Lomb Memorial Drive, Rochester, NY 14623, USA.

^2^Golisano College of Computing and Information Sciences, Rochester Institute of Technology, 20 Lomb Memorial Drive, Rochester, NY 14623, USA.

* To whom correspondence should be addressed. Tel: (+1)585-475-4115; Fax: (+1)585-475-2398; Email: [fxcsbi@rit.edu](mailto:fxcsbi@rit.edu)

Contents

**Supplementary Figures** . . . . . . . . . . . . . . . . . . . . . . . . . . . . . . . . . . . . . . . . . . . . . . . . . . . . . . . . . . . . . . 2

Figure S1 . . . . . . . . . . . . . . . . . . . . . . . . . . . . . . . . . . . . . . . . . . . . . . . . . . . . . . . . . . . . . . . . . . . . . . 2

Figure S2 . . . . . . . . . . . . . . . . . . . . . . . . . . . . . . . . . . . . . . . . . . . . . . . . . . . . . . . . . . . . . . . . . . . . . . 3

Figure S3 . . . . . . . . . . . . . . . . . . . . . . . . . . . . . . . . . . . . . . . . . . . . . . . . . . . . . . . . . . . . . . . . . . . . . . 4

†Joint Authors


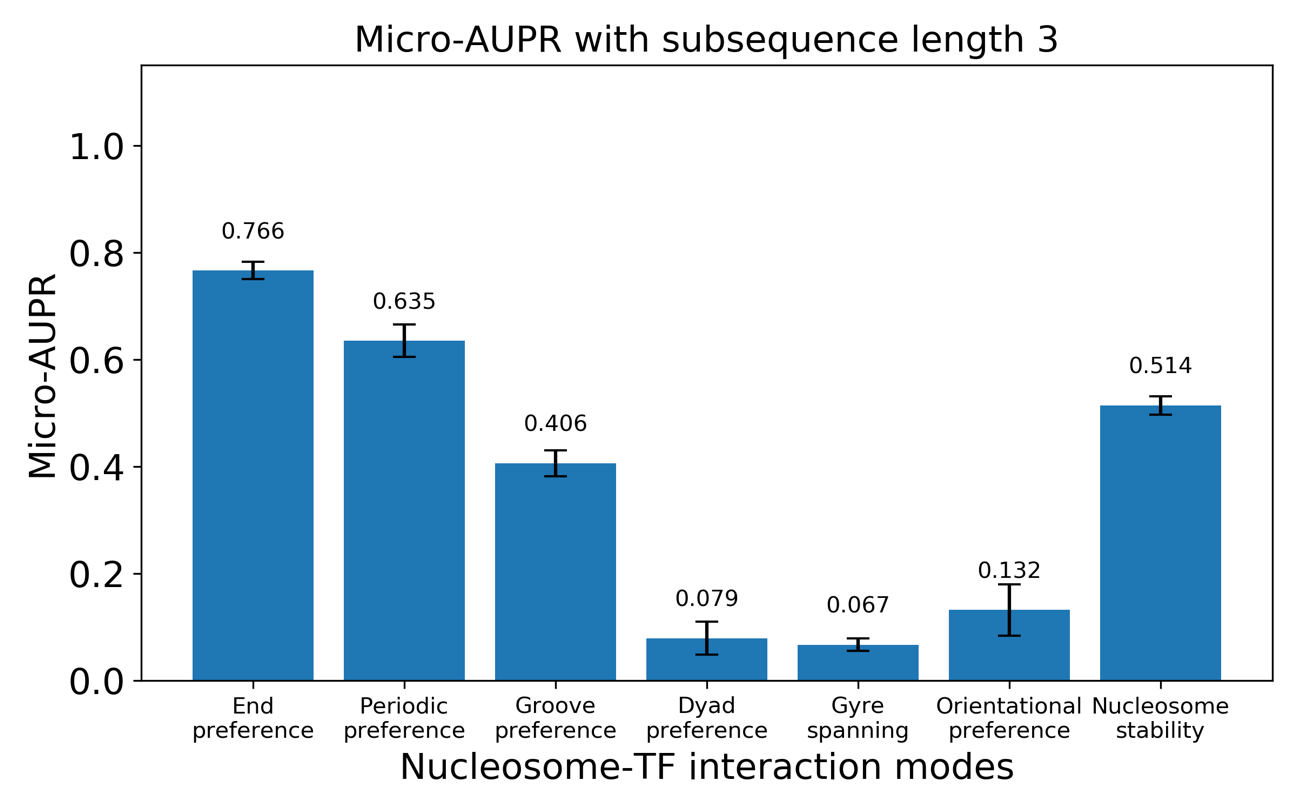


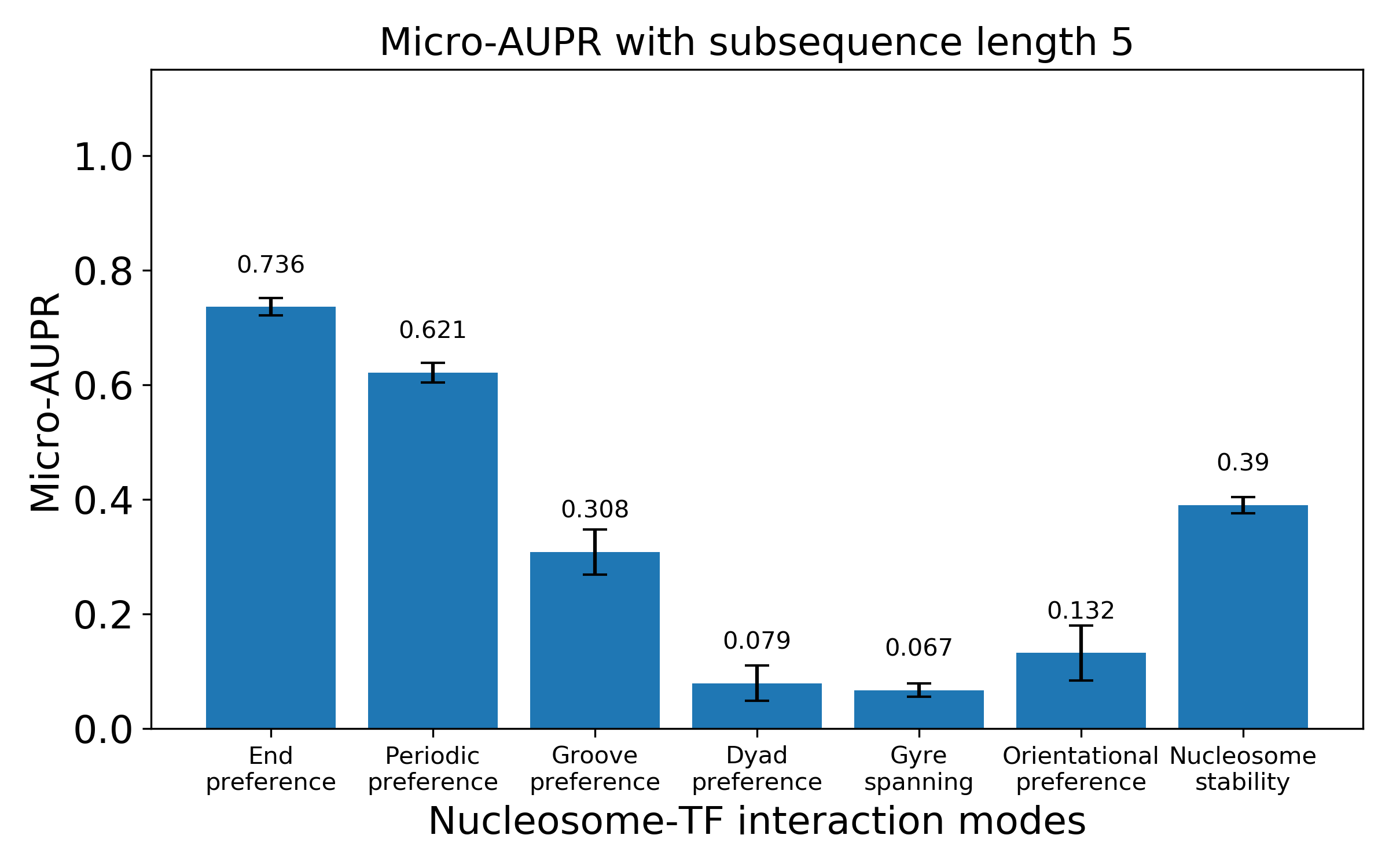


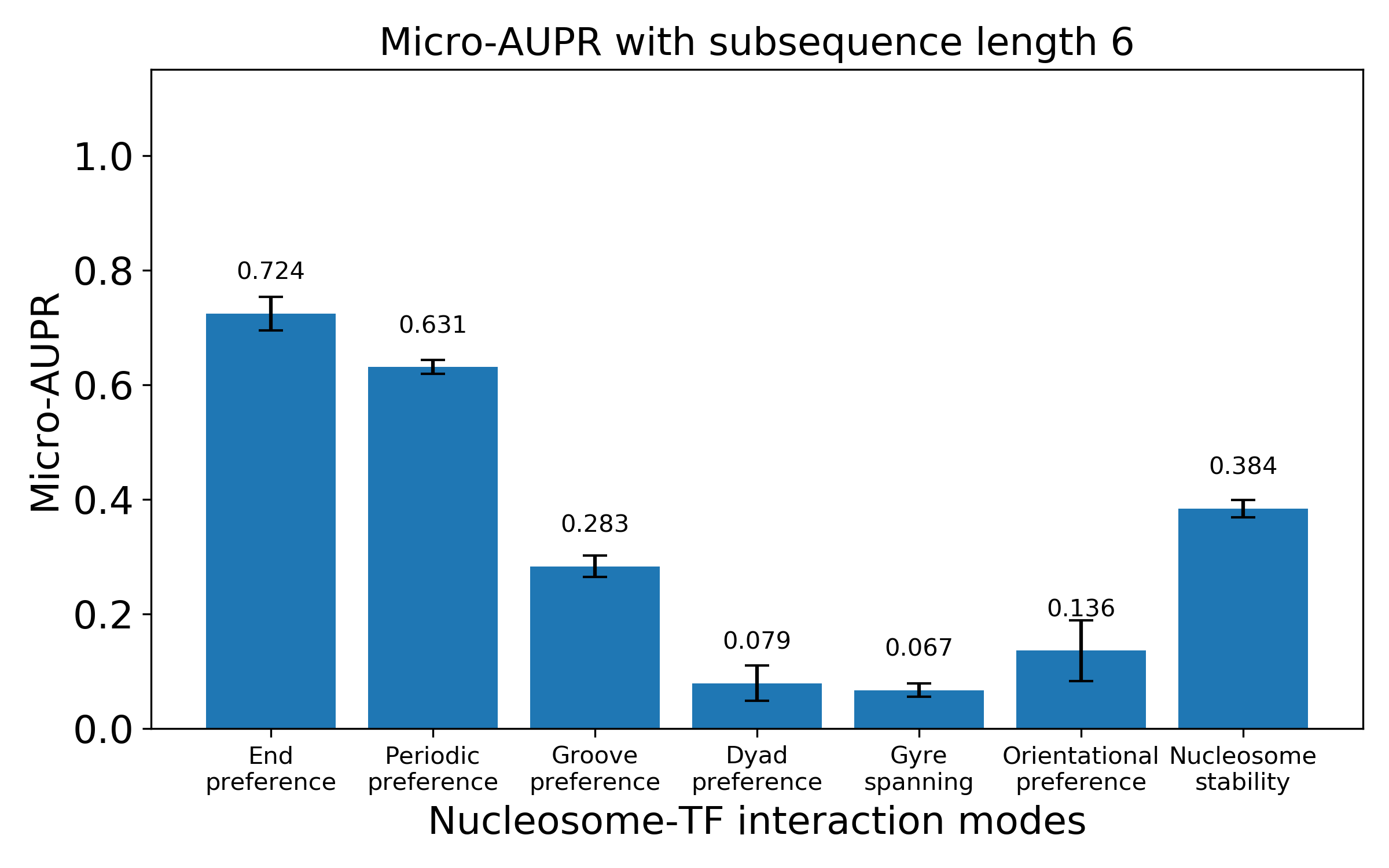

Supplement: Supplementary file 2 — Additional file 2. Supplementary Figures S1 to S3. [file 12859_2021_4093_MOESM2_ESM.docx]
